# Conservation priorities for global marine biodiversity across multiple dimensions

**DOI:** 10.1093/nsr/nwac241

**Published:** 2022-10-31

**Authors:** Huizhong Fan, Mingpan Huang, Youhua Chen, Wenliang Zhou, Yibo Hu, Fuwen Wei

**Affiliations:** Center for Evolution and Conservation Biology, Southern Marine Science and Engineering Guangdong Laboratory (Guangzhou), Guangzhou 511458, China; CAS Key Laboratory of Animal Ecology and Conservation Biology, Institute of Zoology, Chinese Academy of Sciences, Beijing 100101, China; CAS Key Laboratory of Animal Ecology and Conservation Biology, Institute of Zoology, Chinese Academy of Sciences, Beijing 100101, China; University of Chinese Academy of Sciences, Beijing 100049, China; Department of Biodiversity and Ecosystem Services, Chengdu Institute of Biology, Chinese Academy of Sciences, Chengdu 610041, China; Center for Evolution and Conservation Biology, Southern Marine Science and Engineering Guangdong Laboratory (Guangzhou), Guangzhou 511458, China; CAS Key Laboratory of Animal Ecology and Conservation Biology, Institute of Zoology, Chinese Academy of Sciences, Beijing 100101, China; University of Chinese Academy of Sciences, Beijing 100049, China; Center for Evolution and Conservation Biology, Southern Marine Science and Engineering Guangdong Laboratory (Guangzhou), Guangzhou 511458, China; CAS Key Laboratory of Animal Ecology and Conservation Biology, Institute of Zoology, Chinese Academy of Sciences, Beijing 100101, China; University of Chinese Academy of Sciences, Beijing 100049, China

**Keywords:** marine biodiversity, genetic diversity, phylogenetic diversity, conservation priority areas, multiple dimensions

## Abstract

Marine biodiversity plays important roles in ocean ecosystem services and has substantial economic value. Species diversity, genetic diversity and phylogenetic diversity, which reflect the number, evolutionary potential and evolutionary history of species in ecosystem functioning, are three important dimensions of biodiversity. Marine-protected areas have been demonstrated as an effective area-based tool for protecting marine biodiversity, but only 2.8% of the ocean has been fully protected. It is urgent to identify global conservation priority areas and percentage of the ocean across multiple dimensions of biodiversity based on Post-2020 Global Biodiversity Framework. Here, we investigate the spatial distribution of marine genetic and phylogenetic diversity using 80 075 mitochondrial DNA barcode sequences from 4316 species and a newly constructed phylogenetic tree of 8166 species. We identify that the Central Indo-Pacific Ocean, Central Pacific Ocean and Western Indian Ocean harbor high levels of biodiversity across three dimensions of biodiversity, which could be designated as conservation priority areas. We also find that strategically protecting ∼22% of the ocean would allow us to reach the target of conserving ∼95% of currently known taxonomic, genetic and phylogenetic diversity. Our study provides insights into the spatial distribution pattern of multiple marine diversities and the findings would help to design comprehensive conservation schemes for global marine biodiversity.

## Teaser

Assessment of marine biodiversity reveal that strategically protecting approximately 22% of the ocean would allow us to reach the target of conserving approximately 95% of currently known taxonomic, genetic and phylogenetic diversity.

## INTRODUCTION

Biodiversity is the foundation of life on Earth. It provides essential ecological support and services for human survival and development [1]. Species richness (SR), genetic diversity (GD) and phylogenetic diversity (PD) are three important dimensions of biodiversity. SR and GD are two fundamental dimensions of biodiversity and PD is increasingly recognized for its unique values in assessing evolutionary histories of species. Specifically, SR refers to the variety of species or taxonomic groups in a given community or area [2] and enhances the ecosystem functioning [3,4]. GD represents the amount of genetic variability among individuals within a species. It provides the basis for the phenotypic variation and reflects the species’ evolutional potential and ability to respond to the changing environment [5]. PD is defined as the sum of phylogenetic branch lengths for all of the species in an area [6]. It is used as a biodiversity index to measure the timescale of species evolution, identify regions with ancient evolutionary history and predict ecosystem functions and ecosystem diversity [7,8]. Therefore, SR, GD and PD, which reflect the number, evolutionary potential and evolutionary history of species in ecosystem functioning, are three main indices to be measured for biodiversity.

The ocean, comprising the majority of our planet's hydrosphere, is a natural treasure trove of biodiversity [9]. The richness of marine biodiversity plays important roles in maintaining the stability of ocean ecological services [10] and mitigating climate change by promoting carbon sequestration and storage [11]. Moreover, marine biodiversity also has considerable economic value. It feeds millions of people and supports industries that contribute billions of dollars to the global economy [12,13]. Although the spatial distribution of marine SR [14,15] and GD patterns for marine fishes [16] have been investigated, the distribution pattern of PD for global marine animals remains largely unknown.

Human impacts on ocean, particularly overfishing and pollution, are causing the loss of marine biodiversity [17]. Thus, marine-protected areas (MPAs) have been established to conserve the biodiversity and ecosystem of oceans [18]. The MPAs have been confirmed as an effective area-based tool to protect marine biodiversity [19]. However, up to January 2022, only 7.7% of the ocean had been designated as MPAs, of which 2.8% was fully and highly protected [20]. Based on the Post-2020 Global Biodiversity Framework under the Convention on Biological Diversity (CBD) (https://www.cbd.int/), it is urgent to identify new priority areas with high conservation value that are not included in MPAs [21]. Previous efforts to identify global conservation priorities mainly focused on one dimension of marine biodiversity–taxonomic diversity such as SR, endemism and vulnerability [22–24]; other dimensions such as GD and PD are usually neglected [25]. Therefore, there is clearly a need to identify priority areas accounting for multiple dimensions of biodiversity to guarantee that the selected areas have broad biological meaning [26]. Moreover, how much of the sea requires full protection to safeguard marine biodiversity remains challenging. Although earlier studies proposed a quite different percentage of ocean range from 21% to 40% [24,27], these numbers are mainly quantified to conserve marine taxonomic diversity. Therefore, it is necessary to quantify this number from the perspective of protecting multifaceted biodiversity to safeguard more components of marine biodiversity.

Herein, from a macro-genetic perspective, we surveyed the GD and PD of global marine taxa using mitochondrial gene data. We aim (i) to reveal the global distribution patterns of marine PD, (ii) to identify the conservation priority areas across multiple dimensions of marine biodiversity and (iii) to quantitatively evaluate the percentage of ocean areas that needs to be fully protected to safeguard multiple dimensions of marine biodiversity. Our findings would help to design comprehensive conservation schemes for global marine biodiversity and provide a new perspective for the CBD Post-2020 Global Biodiversity Framework.

## RESULTS

### Global distribution of marine GD

**Figure 1.**
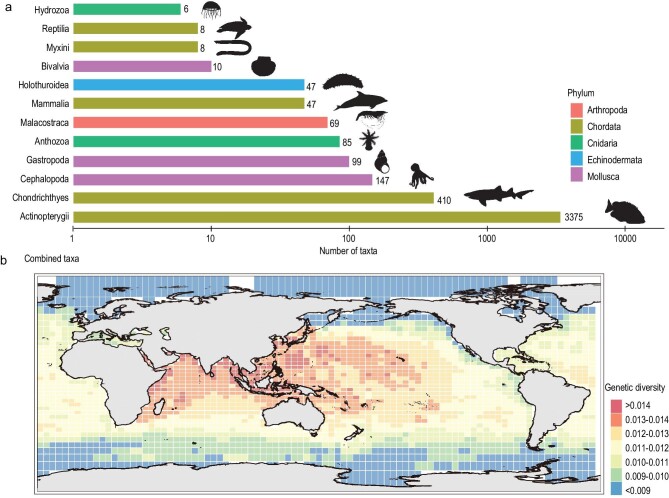
Number of marine taxa used for genetic diversity assessment and global distribution pattern of marine genetic diversity. (a) The number of marine taxa used for genetic diversity assessment. The taxonomic classes are shown from least (top) to the most (bottom). Only taxonomic classes with more than five species are displayed. (b) The spatial pattern of *CO1*-based genetic diversity for global marine species. Species illustrations were taken from http://phylopic.org/ and were available for use under a Public Domain license except that the mammalia illustration credits to Chris huh under Attribution-ShareAlike 3.0 Unported license. 审图号: GS 京 (2023) 0722 号

Taking advantage of publicly available marine sequencing data from the NCBI and BOLD repositories, we obtained a total of 80 075 high-quality mitochondrial *cytochrome oxidase subunit I* (*COI*) sequences from a total of 4316 marine species (Fig. 1a). Using these mitochondrial sequences, we performed species-specific sequence alignment and calculated nucleotide diversity (π) through pairwise comparisons of aligned sequences. To obtain the global distribution map of marine GD, we divided the world ocean map into grid cells measuring 385.9 × 385.9 km and estimated the mean GD of each cell by averaging the GD of species located in the cell. The results showed that the Indo-West Pacific and Western Indian Ocean harbored higher GD, while the regions with low GD were located in the North Atlantic Ocean, Arctic Ocean and Antarctica Ocean (Fig. 1b). We demonstrated that these patterns are robust to variation in the least number of sequences for each species (Supplementary notes), as indicated by the spatial correlation analysis (Supplementary Fig. S1). Moreover, we also proved that the unevenly distributed marine species (Supplementary Fig. S2), different resolution of grid-cell size (Supplementary Fig. S3) and marine species that travel long distances (Supplementary Fig. S4) did not bring substantial bias for the estimation of the global marine GD distribution pattern.

### Global distribution of marine PD

**Figure 2.**
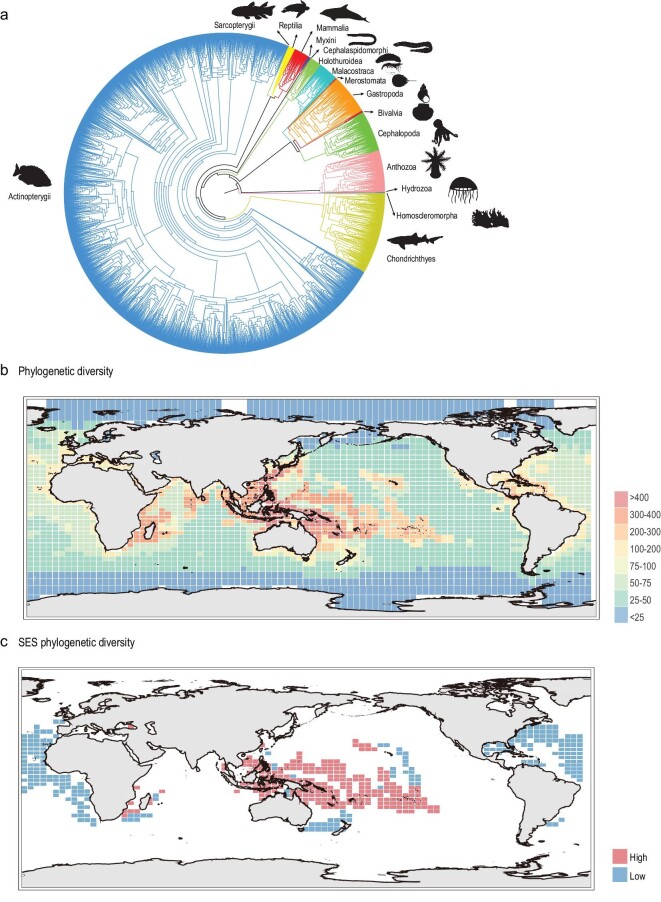
Phylogenetic tree for global marine species and the global distribution patterns of marine phylogenetic diversity. (a) Phylogenetic tree of global marine species based on four mitochondrial genes (*Cytb, Co1, 12S-rRNA* and *Nd1*). (b) Global spatial pattern of marine phylogenetic diversity. (c) The grid cells with the 10% highest (red) and 10% lowest SES-PD value (blue). Species illustrations were taken from http://phylopic.org/ and were available for use under a Public Domain license except that the mammalia illustration credits to Chris huh under Attribution-ShareAlike 3.0 Unported license. 审图号: GS 京 (2023) 0722 号

The species-level PD of global marine species was surveyed based on a newly constructed phylogenetic tree using coding sequences of four mitochondrial genes (*Cytb, Co1, Nd1* and *12S rRNA*). A total of 8166 marine species were assessed for PD with at least one mitochondrial gene sequence. The robustly constructed phylogenetic tree showed that Porifera were a sister group to all other animals, followed by Cnidaria, the Arthropoda formed a clade with Mollusca, Echinodermata and Chordata formed another sister group (Fig. 2a). These results are consistent with the previously published animal tree of life [28]. The distribution map of marine PD was obtained by calculating the PD of marine species within each cell. The results showed that the Central Indo-Pacific Ocean, Western Indian Ocean and Central Pacific Ocean harbored high PD, while the South Indian Ocean, Atlantic Ocean, Eastern Pacific Ocean, Arctic Ocean and Antarctica Ocean showed low levels of PD (Fig. 2b). Additionally, considering that SR is positively correlated with PD in this study (Supplementary Fig. S5) and in many other published studies [29–31], we also calculated the standard effective size of marine phylogenetic diversity (SES-PD) to control for the confounding effect of SR on phylogenetic diversity [32]. In this study, the SES-PD was estimated as the difference in the observed phylogenetic diversity and the mean expected diversity, divided by the standard deviation of the expected PD in 1000 randomizations of the taxa labels. The regions with high SES-PD values mean that they still have high phylogenetic diversity after excluding the effect of taxonomic richness, indicating that a higher proportion of distantly related and anciently diverged taxa could be identified in these regions. The regions with low SES-PD values imply that they have low phylogenetic diversity after excluding the effect of taxonomic richness, indicating that they were the center of recent speciation events and contained recent lineages. The results showed that the areas with the top 10% SES-PD scores were mainly located in the Central Indo-Pacific Ocean and South Pacific Ocean, suggesting that these regions were home to ancestral lineages. In contrast, the areas with the 10% lowest SES-PD values were located in the North Atlantic Ocean (Fig. 2c), indicating that these areas were the centers of recent marine speciation events.

### Relationship between sea-surface temperature and marine biodiversity

To evaluate the relationship between sea-surface temperature and marine biodiversity, we performed spatial analysis between sea-surface temperature and marine SR, GD and PD using a modified *t*-test accounting for spatial autocorrelation. The results showed that sea-surface temperature was significantly correlated with marine SR, GD and PD, indicating that the sea-surface temperature has a positive impact on marine biodiversity (Supplementary Fig. S6).

### Conservation priority areas across three dimensions of marine biodiversity

**Figure 3.**
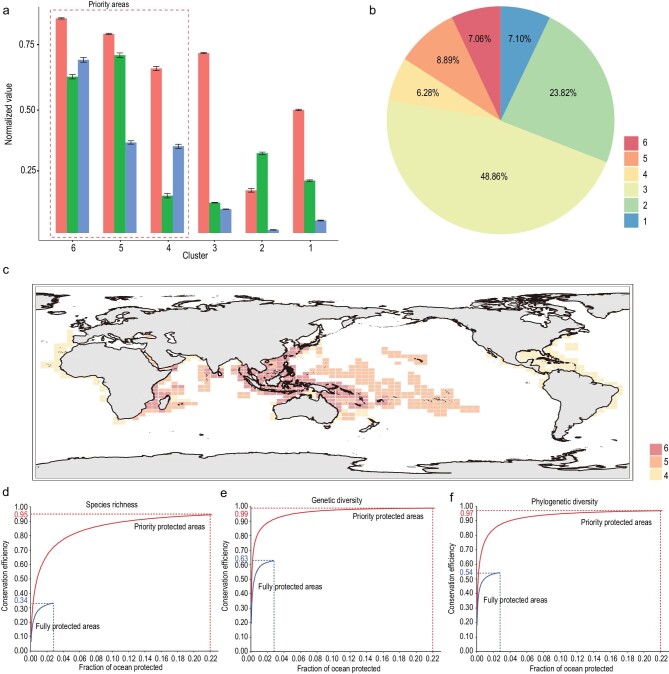
The identification and distribution of priority for conservation across three dimensions of marine biodiversity. (a) The mean values of SR, GD and PD of each cluster. (b) The percentage of world-wide ocean surface covered by each cluster. (c) The spatial distribution of grid cells within priority protected areas; different colors represent different clusters. (d–f) The efficiency of fully protected areas and the priority protected areas in conserving marine (d) species richness, (e) genetic diversity and (f) phylogenetic diversity. 审图号: GS 京 (2023) 0722 号

The priority areas for global marine biodiversity conservation were identified and compared across three key dimensions of biodiversity. Specifically, based on the normalized value of marine SR (Supplementary Fig. S7), GD (Fig. 1b) and SES-PD (Fig. 2b), the grid cells were first clustered into six groups (Supplementary Fig. S8). Then the mean values of three dimensions of marine biodiversity were compared for each cluster and the grid cells in the top three clusters as the conservation priority areas were selected as priority areas (Fig. 3a). We calculated the coverage of each cluster and found that the priority protection areas covered 22.23% of the global ocean surface (Fig. 3b). We mapped the grid cells from the priority areas onto the global world map and found that the areas were mainly located in the Central Indo-Pacific Ocean, Central Pacific Ocean and Western Indian Ocean. In particular, the Indo-Australian Archipelago Ocean and Madagascar island ocean were consistently identified as the largest conservation priority regions (Fig. 3c), indicating that more conservation efforts should be concentrated in these regions.

### Conservation efficiency of currently MPAs and priority areas

We developed a new framework to assess the efficiency of current fully protected areas and priority protection areas in conserving multifaceted biodiversity components. The results showed that current fully protected areas perform poorly in terms of protecting multiple dimensions of marine biodiversity. In detail, the fully protected areas conserved only 34%, 63% and 54% of currently known taxonomic, genetic and PD, which are significantly lower than the percentages protected by randomly selected areas (Supplementary Fig. S9). In contrast, the priority areas that we identified could conserve 95%, 99% and 97% of taxonomic, genetic and PD, respectively (Fig. 3d–f), which are significantly higher than the percentages obtained by randomly selected areas (Supplementary Fig. S10). These results could help to quantify the exact percentage of global marine areas that needs to be fully and highly protected. Specifically, we could conserve 95% of currently inventoried multidimensional biodiversity (taxonomic, genetic and phylogenetic) by strategically protecting ∼22% of the global ocean (Fig. 3d–f).

## DISCUSSION

This is the first multiple survey of species, genetic and phylogenetic diversities for global marine species and the results reveal that the regions located in the Indo-West Pacific harbored the higher marine SR (Supplementary Fig. S7), GD (Fig. 1b) and PD (Fig. 2b), supporting previous studies which revealed that this region was a SR hotspot for marine animals and plants [15,33]. Previous studies have proposed four hypotheses to explain the high level of species biodiversity in this region: centers of origin [34], centers of accumulation [35], centers of overlap [36] and centers of survival [37]. A study revealed that tropical reef biodiversity hotspots have changed from the Western Tethys to Indo-Pacific areas since the Eocene, supporting the centers-of-survival hypothesis [38]. In this study, we found that the Indo-West Pacific has high SES-PD, indicating that ancestral lineages can survive and thrive in this region (Fig. 2c). This provides strong evidence for the centers-of-survival hypothesis, which suggests that this region is a refuge shelter for many ancestral species.

MPAs have been demonstrated to be one of the most effective tools for restoring marine biodiversity and ecosystem services [18]. The requirement to increase the coverage of MPAs has been already recognized in the CBD Post-2020 Global Biodiversity Framework. Previous studies focused on the areas featuring high marine taxonomic diversity [22–24], which might neglect the conservation of areas containing species with high evolutionary potential and older evolutionary histories. In this study, we captured the priority areas for marine animals across three dimensions of biodiversity—taxonomic, genetic and phylogenetic. The results revealed that the conservation priority areas are mainly located in the Central Indo-Pacific Ocean, Central Pacific Ocean and Western Indian Ocean (Fig. 3c), suggesting that these regions should receive special conservation attention.

The percentage of ocean that requires to maximally protecting marine biodiversity is a main CBD target. During the recent 15th meeting of the Conference of the Parties (COP15) held in Kunming, the CBD declared that ≥30% of global sea areas should be protected by 2030 [39]. In this study, from the perspective of protecting multifaceted biodiversity components, our results showed that strategically protecting ∼22% of the global ocean would allow us to reach the target of conserving ∼95% of currently known taxonomic, genetic and PD (Fig. 3c–e). These results may provide an insight in the context of setting global marine biodiversity conservation targets. Of course, science-based expansion of MPAs should not only consider the conservation of biodiversity, other important factors such as food provision and carbon storage should also be taken into consideration in the future [27]. In addition, although PD has the potential to identify and prioritize species in need of protection and improve the spatial planning of conservation areas, it may not be able to forecast the functional diversity (FD) of species because it still depends on many assumptions, uncertainties and varying messages [40]. To better conserve biodiversity, FD that reflects the ecological, morphological and physiological strategies of species [41] should also be taken into consideration. Therefore, to design effective conservation planning, multiple dimensions of biodiversity including taxonomic, genetic, phylogenetic and functional diversities should be incorporated to ensure the biodiversity persistence in a changing world.

## METHODS

### Estimation of marine GD

The mitochondrial CO1 coding sequences for marine species were retrieved from GenBank (www.ncbi.nlm.nih.gov/genbank) and the BOLD database (www.boldsystems.org). For each marine species, we selected the corresponding sequences from the database and performed sequence alignment analysis using MUSCLE software with default parameters [42]. Only the pairwise alignments whose sequence overlaps were >60% and sequence differences were <10% were used to calculate GD. The GD of each species was defined and calculated following Miraldo *et al.* [43].

To obtain the distribution pattern of global marine GD at a finer scale, we divided the world ocean map into grids measuring 385.9 × 385.9 km representing an area of 148 953 km^2^. Grid cells including coastal habitat in which ocean area accounted for <50% of the total area were excluded from the analysis. The GD of each cell was calculated by averaging Π across all the species located in the cell, which was mathematically defined by:


\begin{eqnarray*} GD = \frac{1}{S}\sum\limits_{p = 1}^S \prod \end{eqnarray*}

where *S* is the number of species in the cell.

### Estimation of marine PD

We constructed the phylogenetic tree of global marine species based on four mitochondrial genes (*Cytb, Co1, 12S-rRNA* and *Nd1*). We first aligned the coding sequences of each gene using MAFFT software with default parameters [44] and trimmed the poorly aligned sites at the start and end of the sequence. Then, we imported the aligned results of four genes into SequenceMatrix software [45] and constructed a supermatrix with gaps regarded as missing data. Finally, we constructed a phylogenetic tree of global marine species using RAxML 8.2.12 [46] with the ASC_GTRGAMMA model and 1000 bootstrap replicates. The species *Oscarella Microlobata* and *Pseudocorticium Jarrei* from *Homoscleromorpha* were used as the outgroups. We calculated Faith's phylogenetic diversity [6] and SES-PD [32] using the ‘picante’ package [47] in R software.

### Collection of global marine species distribution, sea-surface temperature and MPA data

We directly derived the distribution data of global marine species in vectorized shapefile format from the IUCN spatial database (www.iucnredlist.org/resources/spatial-data-download). The global map was from China Ministry of Natural Resources (http://bzdt.ch.mnr.gov.cn/index.html). The sea-surface temperature data sets were collected from MARSPEC database [48]. The spatial information on global MPAs was collected from the World Database on Protected Areas available at http://protectedplanet.net/.

### Identification of priority areas for marine biodiversity conservation

In this study, priority areas were selected based on three important dimensions of marine biodiversity: SR, GD and PD. To obtain the regions with highest levels of biodiversity across multiple dimensions, we introduced a k-means clustering method [49] to classify the grid cells. In detail, the SR, GD and SES-PD values of each grid cell were first normalized from zero to one using the min–max normalization method. Then, the optimal number of clusters was determined using the fviz_nbclust function implemented in the factoextra R package. Finally, the k-means method was used to cluster the grid cells and the priority areas for marine conservation were selected based on the normalized marine biodiversity values.

### Assessing the effectiveness of conserving multifaceted biodiversity components

To assess the effectiveness of the proposed priority areas in protecting multifaceted biodiversity components, we used a biodiversity-preservation cumulative curve with 95% confidence interval, which is to randomly sample an increasing number of grid cells from all the available grid cells with 1000 replicates. This randomized biodiversity-preservation curve is applied to the assessments of conservation effectiveness of SR, GD and PD in fully protected areas versus proposed priority areas. The detailed steps to obtain species preservation cumulative curves are displayed in the Supplementary notes.

## Supplementary Material

nwac241_Supplemental_File

## Data Availability

All data and code reported in this paper have been deposited in Github database (https://github.com/fanhuizhong).
