# Supplementary material for: Conservation priorities for global marine biodiversity across multiple dimensions
Source: Natl Sci Rev. 2022 Oct 31;10(6):nwac241. doi: 10.1093/nsr/nwac241 (PMC10171637; doi:10.1093/nsr/nwac241)
Supplement: nwac241_Supplemental_File [file nwac241_supplemental_file.docx]

Supplementary Information for

**Conservation priorities for marine biodiversity across multiple dimensions**

Huizhong Fan^1,2*^, Mingpan Huang^2*^, Youhua Chen^3*^, Wenliang Zhou^1^, Yibo Hu^2,4^, Fuwen Wei^1,2,4#^

^1^Southern Marine Science and Engineering Guangdong Laboratory (Guangzhou), Guangdong, 511458, China.

^2^CAS Key Laboratory of Animal Ecology and Conservation Biology, Institute of Zoology, Chinese Academy of Sciences, Beijing, 100101, China.

^3^Chengdu Institute of Biology, Chinese Academy of Sciences, Chengdu, 610041, China.

^4^University of Chinese Academy of Sciences, Beijing, 100049, China.

*These authors contributed equally to this work.

^#^Correspondence should be addressed to Fuwen Wei (weifw@ioz.ac.cn).

**This Supplementary Information includes:**

**Supplementary Notes**

**Supplementary Figures 1-10**

**Collection of marine genetic diversity data**

We used following steps to retrieve corresponding mitochondrial *CO1* coding sequences for marine species from GenBank (www.ncbi.nlm.nih.gov/genbank) and the BOLD database (www.boldsystems.org). First, we searched the GenBank database using the rules “(CO1[All Fields] OR "COX1"[All Fields] OR "COI"[All Fields] OR "COXI"[All Fields] OR "cytochrome oxidase subunit 1"[All Fields]) AND (species Latin name [Organism])” and downloaded the corresponding gene sequences. Then, we downloaded the marine *CO1* sequences from the BOLD database using the application-platform interface (API) (<http://www.boldsystems.org/index.php/resources/api>). We extracted and integrated the union coding sequences of these two databases based on the header of the sequence. Finally, we filtered the data using the following criteria: 1) short genes whose coding sequence length was < 300 bp were discarded, and 2) species with fewer than three sequences were also removed.

**Calculation genetic diversity** **for each marine species**

The genetic diversity of each species was defined following Mirado *et al.* [1] and mathematically calculated using the following steps. We first calculated the nucleotide diversity as the average number of variable sites in each pairwise sequence comparison. It is mathematically defined by:

Where k_ij_ is the number of different nucleotides between sequence i and sequence j, and $\left(\frac{n}{2}\right)$ represents the number of pairwise comparisons. Because the value of $\Pi$ depends on the length of sequence *(m)*, it should divided by m to obtain the obtain the nucleotide diversity. However, as the pairwise sequences comparison has a different length, dividing $\Pi$ by m would give incorrect values of nucleotide diversity. To account for this, we divided the k_ij_ by the length of sequence in each pairwise comparison instead. It is mathematically defined by:

where k_ij_ is the number of different nucleotides between sequence i and sequence j, m_ij_ is the number of shared bases between sequence i and sequence j, and $\left(\frac{n}{2}\right)$ is the number of pairwise comparisons.

**Evaluation of the robust of marine genetic diversity**

To evaluate the impact of the number of sequences for each species on the estimated genetic diversity, we selected and reanalyzed different parts of the original data and compared the results based on different sequence numbers via spatial correction analysis. In detail, we selected and retained the species with at least four, five and ten sequences and recalculated the genetic diversity of each grid cell again using the above steps. Then, we used a modified t test accounting for spatial autocorrelation [2,3] to perform the spatial correlation analysis and check the consistency among different results based on species with at least four, five and ten sequences. The results showed that marine genetic diversity patterns are robust to variation in the least number of sequences for each species (Fig. S1). In this study, we presented a global genetic map based on species with at least four sequences because marine genetic diversity is not systematically affected by the sequence number of each species (Fig. 1b). All of the analyses were performed at a 385.9 km × 385.9 km grid cell resolution.

To test whether unevenly distributed species would bring bias for the estimation of marine genetic diversity, we randomly selected 50, 70 and 100 species for each grid cell and recalculated the global marine genetic diversity distribution maps. We checked the consistency among different results by performing the spatial correlation analysis between results based on different number of species in each grid cell and that based on all of the species in each grid cell. The results showed that the global distribution patterns of marine genetic diversity map based on different number of species in each grid cell remain similar (Fig. S2), indicating that the unevenly sampling distribution did not bring substantial bias for the estimation of marine genetic diversity distribution.

To determine whether different resolution of grid cell size bring bias for the estimated genetic diversity, we recalculated the marine genetic diversity based on different resolutions of grid cells and compared these results with that based on 385.9km x 385.9 km grid cells via spatial correction analysis. In detail, we first divided the world ocean map into three nested equal area grids with cell sizes of 96.5km x 96.5 km, 193 km x 193 km and 771 km x 771 km, respectively. Then we recalculated the marine genetic diversity for each grid cell with different size and checked the consistency between the genetic diversity results based on different resolution of grid cells and that based on 385.9km x 385.9 km grid cells by performing spatial correlation analysis. The results showed that the distribution patterns of the genetic diversity remain similar, indicating that the marine genetic diversity is not systematically affected by the resolution of grid cell size in this study (Fig. S3).

To determine whether the marine species that travel long-distance may bring bias for the estimated genetic diversity, we collected a total of 718 long-distance marine species including migratory fishes, cetaceans and turtles from published literature [4]. We removed these long-distance migrated species from our dataset and recalculated the marine genetic diversity. We compared the results between the genetic diversity results based on these two datasets. The results showed that a significant correlation for these two genetic diversity distribution patterns was identified (Fig. S4), indicating that marine genetic diversity is not systematically affected by long-distance movement.

**Collection of marine phylogenetic diversity data**

We used four mitochondrial genes (*Cytb*, *Co1*, *12S-rRNA* and *Nd1*) to construct the phylogenetic tree of global marine species. The sequences of these four genes were obtained using the following steps. First, we downloaded the available mitochondrial reference genomes from the GenBank database and extracted the corresponding coding sequences. Then, we directly downloaded the coding sequences of the remaining species from the GenBank database using the species Latin name and corresponding gene name. In particular, the coding sequences for the mitochondrial genes *Cytb* and *Co1* in the BOLD database were also collected. Finally, we excluded short genes whose sequence length was less than 300 bp and used sequences whose length was similar to that of the corresponding gene to construct the phylogenetic tree. After these steps, we had data for a total of 8,166 marine species, including 350 *Cnidaria*, 688 *Mollusca*, 168 *Arthropoda*, 91 *Echinodermata* and 6,869 *Chordata* species. Our dataset covered 15 classes, 88 orders, 492 families and 2,289 genera.

**Calculation of phylogenetic diversity and SES-PD**

Based on the newly constructed phylogenetic tree and marine species distribution data, we calculated Faith’s phylogenetic diversity [5] and SES-PD [6] using the “picante” package [7] in R software. Faith’s phylogenetic diversity is the sum of all phylogenetic branch lengths within a grid cell. SES-PD was calculated because phylogenetic diversity is significantly correlated with species richness. For each grid cell, we first obtained a null distribution of expected phylogenetic diversity values by shuffling the marine taxa labels across the tips of the tree 1,000 times. SES-PD was calculated by dividing the difference between the observed (PD_observed_) and expected phylogenetic diversity (PD_random_) values by the standard deviation of the null distribution (sd.(PD_random_)), which was mathematically defined by:

$$SES-PD=\frac{PD_{observed}-PD_{random}}{s.d.(PD_{random})}$$

**Collection of global marine species distribution, sea-surface temperature and MPA data**

We directly derived the distribution data of global marine species in vectorized shapefile format from the IUCN spatial database ([www.iucnredlist.org/resources/spatial-data-download](http://www.iucnredlist.org/resources/spatial-data-download)) and rasterized them into a grid system with a 385.9 km × 385.9 km resolution. The resultant rasterized maps were double-checked to confirm that they were actually mapped to the original distribution maps. The global map was from China Ministry of Natural Resources (http://bzdt.ch.mnr.gov.cn/index.html). In total, we collected distribution maps for 14,562 marine species. After matching the marine genetic and phylogenetic data, we finally used 4,086 and 7,692 species for the global marine genetic diversity and phylogenetic diversity analyses. The sea-surface temperature data were collected from MARSPEC database [8]. Because the marine genetic diversity and phylogenetic diversity were assessed using a grid cell of 385.9km x 385.9km resolution, sea-surface temperature data were averaged within each gird cell as the variable’s value for each grid cell. The spatial information on global MPAs was collected from the World Database on Protected Areas (WDPA) available at <http://protectedplanet.net/>. As the original database contained both terrestrial and ocean PAs, we first selected ocean PAs based on the reported marine area value. Then we merged the union regions of overlapping MPAs and divided them into 385.9 km × 385.9 km grid cells. Finally, a total of 4,422 MPAs covering a 25,122,343 km^2^ surface were retained in the subsequent analysis.

**Assessing the** **effectiveness of conserving multifaceted biodiversity components**

To assess the effectiveness of conserving species in selected priority sites, suppose there are S species in z grid cells and we sample a subset $z'$ of the z grid cells; then, the percentage of species conserved is evaluated as:

$$SD\%=\frac{S(z')}{S}\times100\%$$

where $S(z')$ denotes the number of species found in the $z'$ grids. In practice, to draw the species preservation curve with 95% confidence intervals, we randomly sample $z'$ grid cells from all the available grid cells with 1,000 replicates. This randomization process is applied to the following assessments of conservation effectiveness of phylogenetic diversity and genetic diversity.

To assess the effectiveness of conserving phylogenetic diversity in selected priority sites, suppose the S species in the z grid cells constitute a phylogenetic tree with internal branch set B. The total phylogenetic diversity is calculated as:

$$PD(S)=\sum_{i\in B} L_{i}$$

where $L_{i}$ denotes the length of a specific branch in the tree. When sampling $z'$ grid cells, the phylogenetic diversity that can be represented is given by:

$$PD(S(z'))=\sum_{i\in B(z')} L_{i}$$

where $B(z')$ represents a subtree of the original tree with branch set B. This subtree has tip species from those species found in the $z'$ grid cells, and its root is identical to that of the original tree. As such, the percentage of phylogenetic diversity conserved can be evaluated as:

$$PD\%=\frac{PD(S(z'))}{PD(S)}\times100\%$$

Finally, for genetic diversity, we developed an additive partitioning framework to assess the effectiveness of the priority areas identified. To be more specific, we first defined region-level genetic diversity for a single species *i*, which is simply measured as Nei’s genetic diversity index based on all the aligned DNA sequences with a number of $n_{i}$ summed from a set of z local grid cells ($n_{i}=\sum_{j\in z} n_{\mathrm{ij}}$, where $n_{\mathrm{ij}}$ represents the number of DNA sequences for species i in the local grid site j).

$$\Pi_{i}=\frac{1}{n_{i}(n_{i}-1)/2}\sum_{s=1}^{n_{i}-1} \sum_{r=s+1}^{n_{i}} \frac{k_{\mathrm{sr}}}{m}$$

where m is the length of each aligned DNA sequence. Accordingly, we define local-level within-cell genetic diversity for species *i* in a specific grid cell *j* as:

$\Pi_{i,j}=\frac{1}{n_{\mathrm{ij}}(n_{\mathrm{ij}}-1)/2}\sum_{s=1}^{n_{\mathrm{ij}}-1} \sum_{r=s+1}^{n_{\mathrm{ij}}} \frac{k_{\mathrm{sr}}}{m}$,

and between-cell genetic diversity for species *i* in a pair of grid cells *j* and *l* as:

$$\Pi_{i,jl}=\frac{1}{n_{\mathrm{ij}}n_{\mathrm{il}}}\sum_{s=1}^{n_{\mathrm{ij}}} \sum_{r=1}^{n_{\mathrm{il}}} \frac{k_{\mathrm{sr}}}{m}$$

We can prove that region-level genetic diversity can be partitioned into a local within-grid genetic diversity component and a local between-grid genetic diversity component, weighted by the relative abundance of the sequences. In a statistical sense, the additive partitioning framework can be written as

$${\frac{n_{i}(n_{i}-1)}{2}\Pi}_{i}=\sum_{i=1}^{n_{i}-1} \sum_{j=i+1}^{n_{i}} \frac{k_{\mathrm{ij}}}{m}=\sum_{j=1}^{m} \frac{n_{\mathrm{ij}}(n_{\mathrm{ij}}-1)}{2}\Pi_{i,j}+\sum_{j=1}^{m} \sum_{l\neq j} n_{\mathrm{ij}}n_{\mathrm{il}}\Pi_{i,jl}$$

Therefore,

$$\Pi_{i}=\sum_{s=1}^{n_{i}-1} \sum_{r=s+1}^{n_{i}} \frac{k_{\mathrm{sr}}}{m}=\sum_{j\in z} \frac{n_{\mathrm{ij}}(n_{\mathrm{ij}}-1)}{n_{i}(n_{i}-1)}\Pi_{i,j}+\sum_{j\in z} \sum_{l\in z,l\neq j} \frac{n_{\mathrm{ij}}n_{\mathrm{il}}}{n_{i}(n_{i}-1)/2}\Pi_{i,jl}$$

This is our spatial additive framework for partitioning regional genetic diversity into local components. This equation implies that spatial genetic diversity includes within-site components and between-site components. In our study, this equation allows us to quantify the effectiveness of conserving a proportion of priority sites for preserving regional genetic diversity. To do so, suppose that we sample a subset $z'$ of the z grid cells; the region-level genetic diversity that can be represented by these subsets of cells can be given as

$$\Pi_{i}(z’)=\sum_{j\in z'} \frac{n_{\mathrm{ij}}(n_{\mathrm{ij}}-1)}{n_{i}(n_{i}-1)}\Pi_{i,j}+\sum_{j\in z'} \sum_{l\in z',l\neq j} \frac{n_{\mathrm{ij}}n_{\mathrm{il}}}{n_{i}(n_{i}-1)/2}\Pi_{i,jl}$$

As such, the proportion of region-level genetic diversity of species *i* conserved by this set $z'$ of priority sites can be computed as

$${\mathrm{GD}_{i}\%=\Pi}_{i}(z’)/\Pi_{i}(z)\times100\%$$

At both regional and local levels, we actually need to average the genetic diversities of all the species to obtain an overall genetic diversity value. As such, the proportion of region-level genetic diversity of all the species (total of S species as assumed above) conserved by the set $z'$ of priority sites can be computed as

$$GD\%=\frac{\sum_{i=1}^{S} \Pi_{i}(z')/S}{\sum_{i=1}^{S} \Pi_{i}(z)/S}\times100\%=\frac{\sum_{i=1}^{S} \Pi_{i}(z')}{\sum_{i=1}^{S} \Pi_{i}(z)}\times100\%$$

**
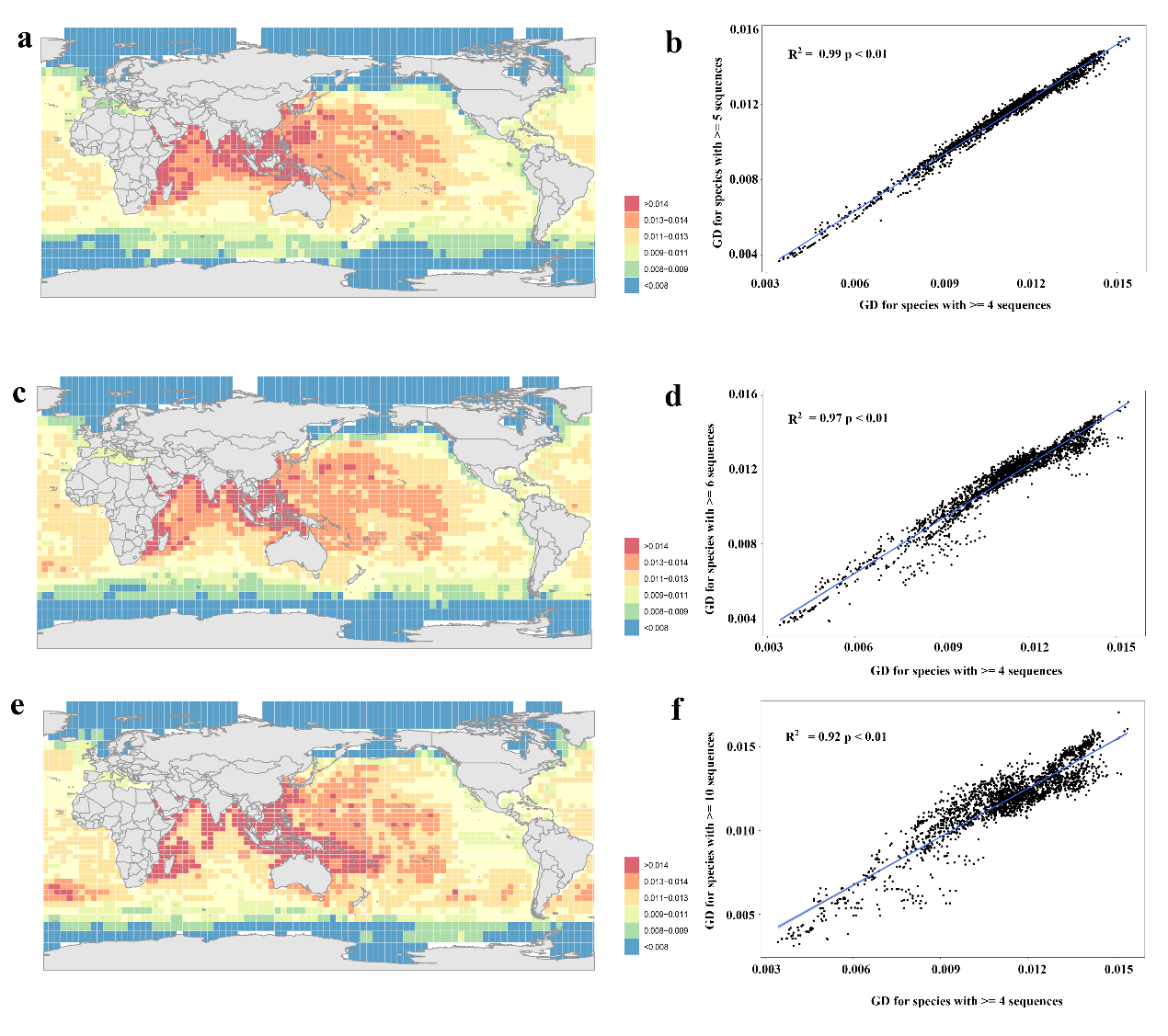
**

**Fig. S1 The effect of sequence number of each species on global marine genetic diversity map. (**a, c, e) Spatial distribution pattern of genetic diversity calculated for those species with more than 5 sequences (a) 6 sequences and (c) 10 sequences (e). (b, d, f) The spatial correlation between the genetic diversity for species with more than 4 sequences (Fig. 1b) and genetic diversity for species with more than 5 sequences (b), 6 sequences (d) and 10 sequences (f).


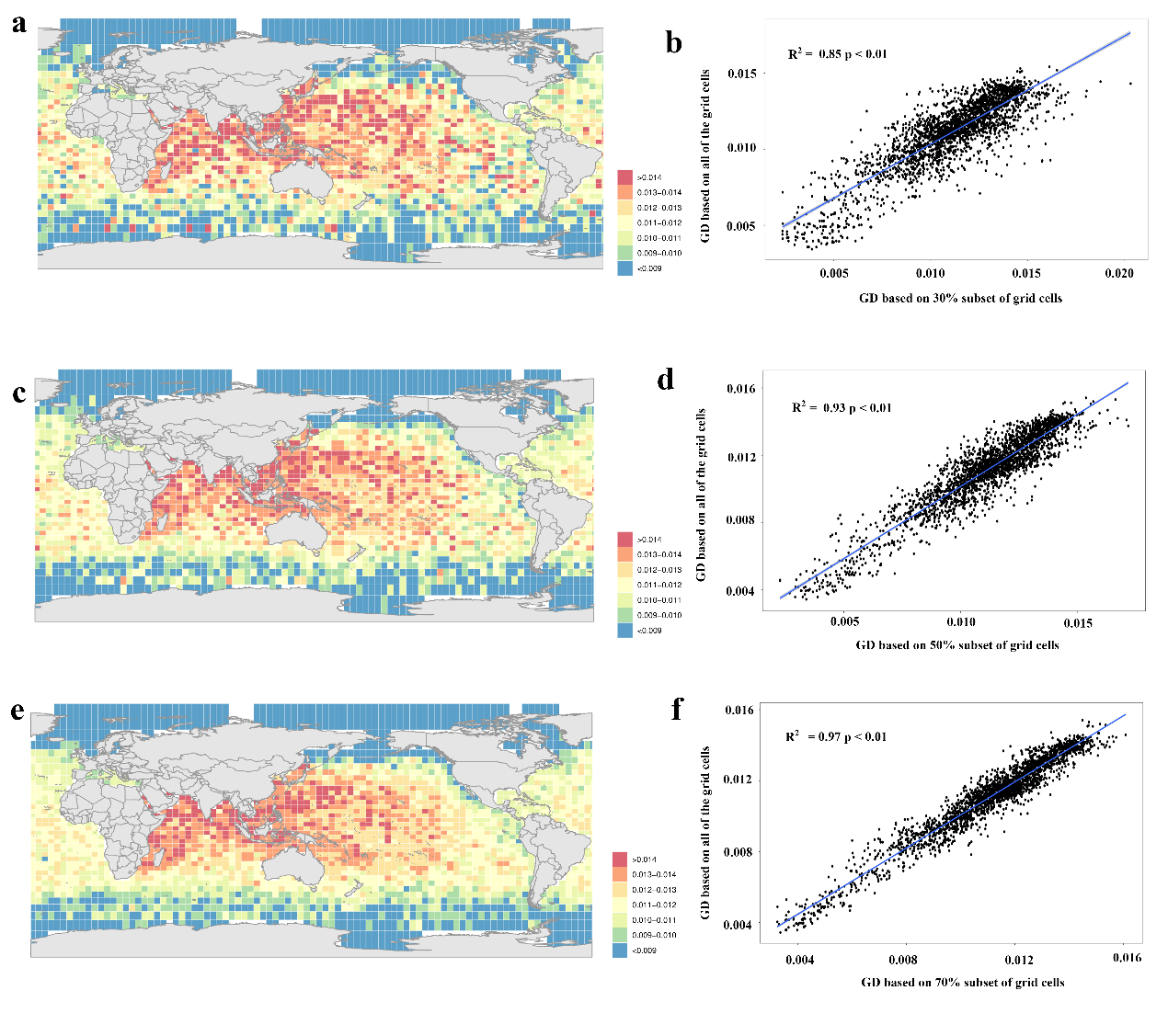


**Fig. S2 The effect of unevenly distributed species on global marine genetic diversity map. (**a, c, e) Spatial distribution pattern of genetic diversity based on randomly selected 30% (a) 50% (c) and 70% (e) subset of grid cells. (b, d, f) The spatial correlation between the genetic diversity based on 30% (b) 50% (d) and 70% (f) subset of grid cells and genetic diversity based on all of the grid cells (Fig. 1b) .


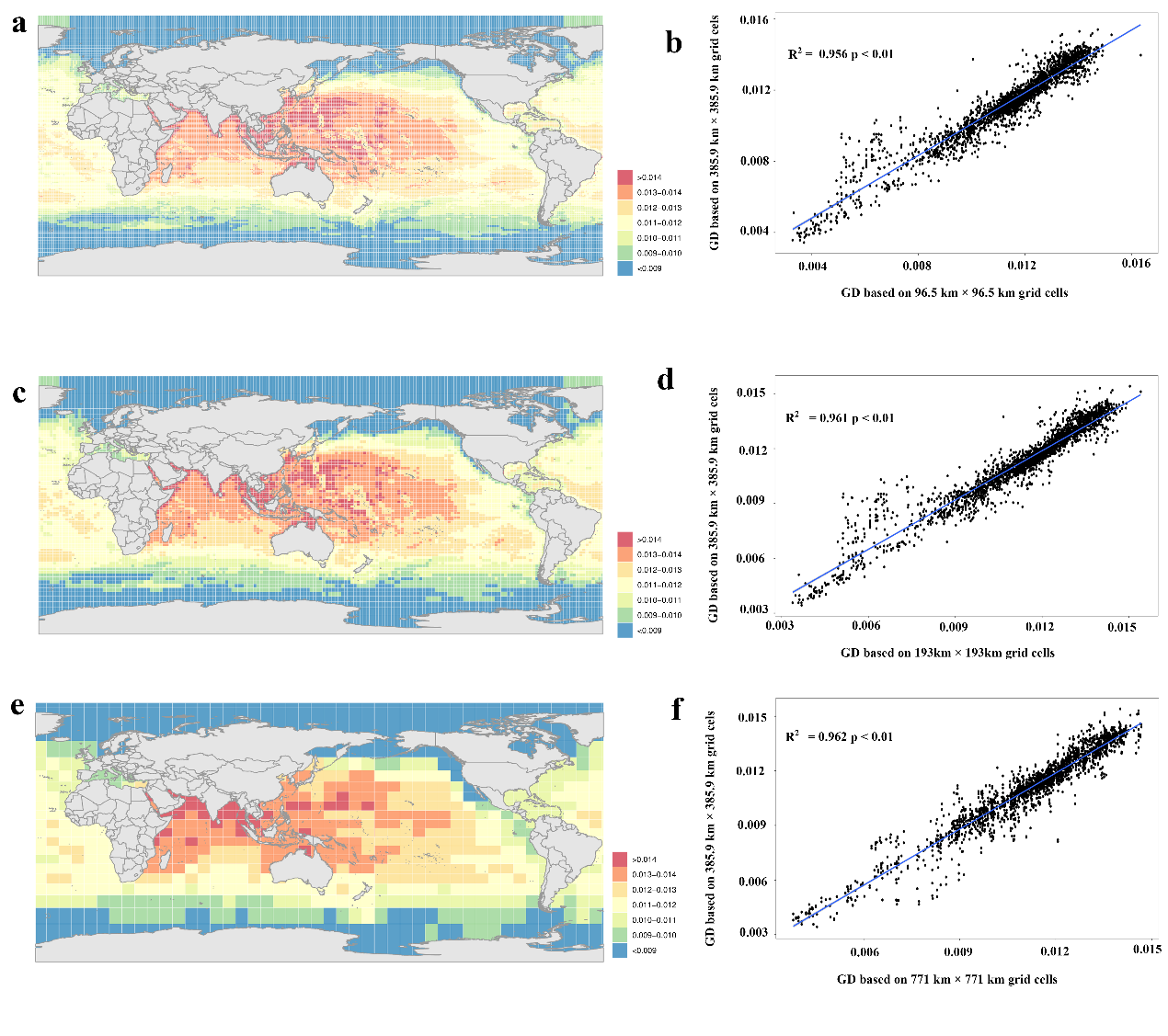


**Fig. S3 The effect of different resolution of grid cells on global marine genetic diversity map. (**a, c, e) Spatial distribution pattern of genetic diversity based on 96.5km × 96.5 km (a) 193km × 193 km (c) and 771km × 771 km (e) grid cells. (b, d, f) The spatial correlation between the genetic diversity based on 96.5km × 96.5 km (b) 193km × 193 km (d) and 771km × 771 km (f) grid cells and genetic diversity based on 385.9 × 385.9 grid cells (Fig. 1b) .


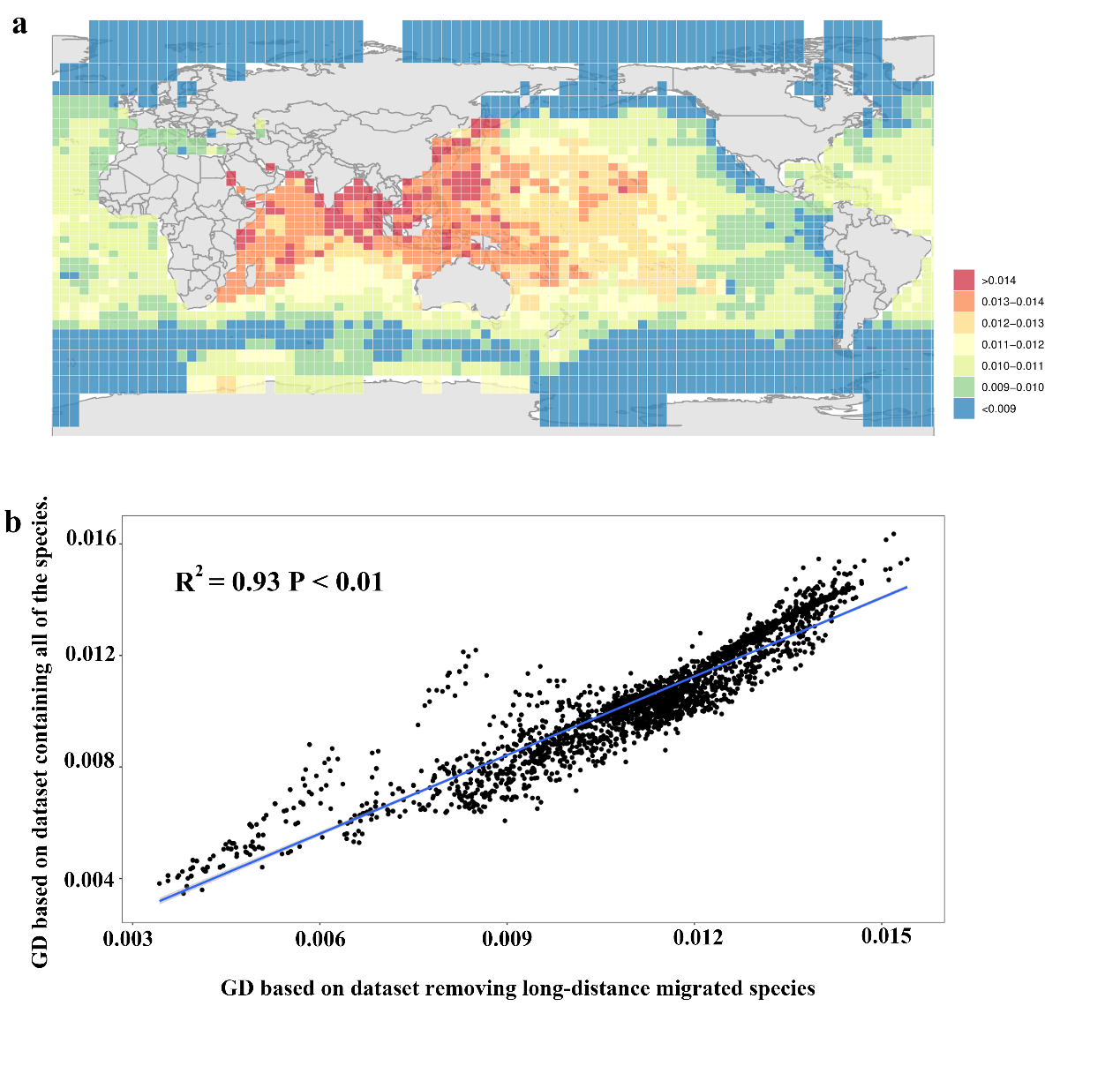


**Fig. S4 The effect of long distance movement marine species on global marine genetic diversity map.** a) Spatial distribution pattern of genetic diversity based on species distribution dataset removing long-distance migrated species. b) The spatial correlation between genetic diversity based on the dataset removing long-distance migrated species and the dataset containing all of the marine species.


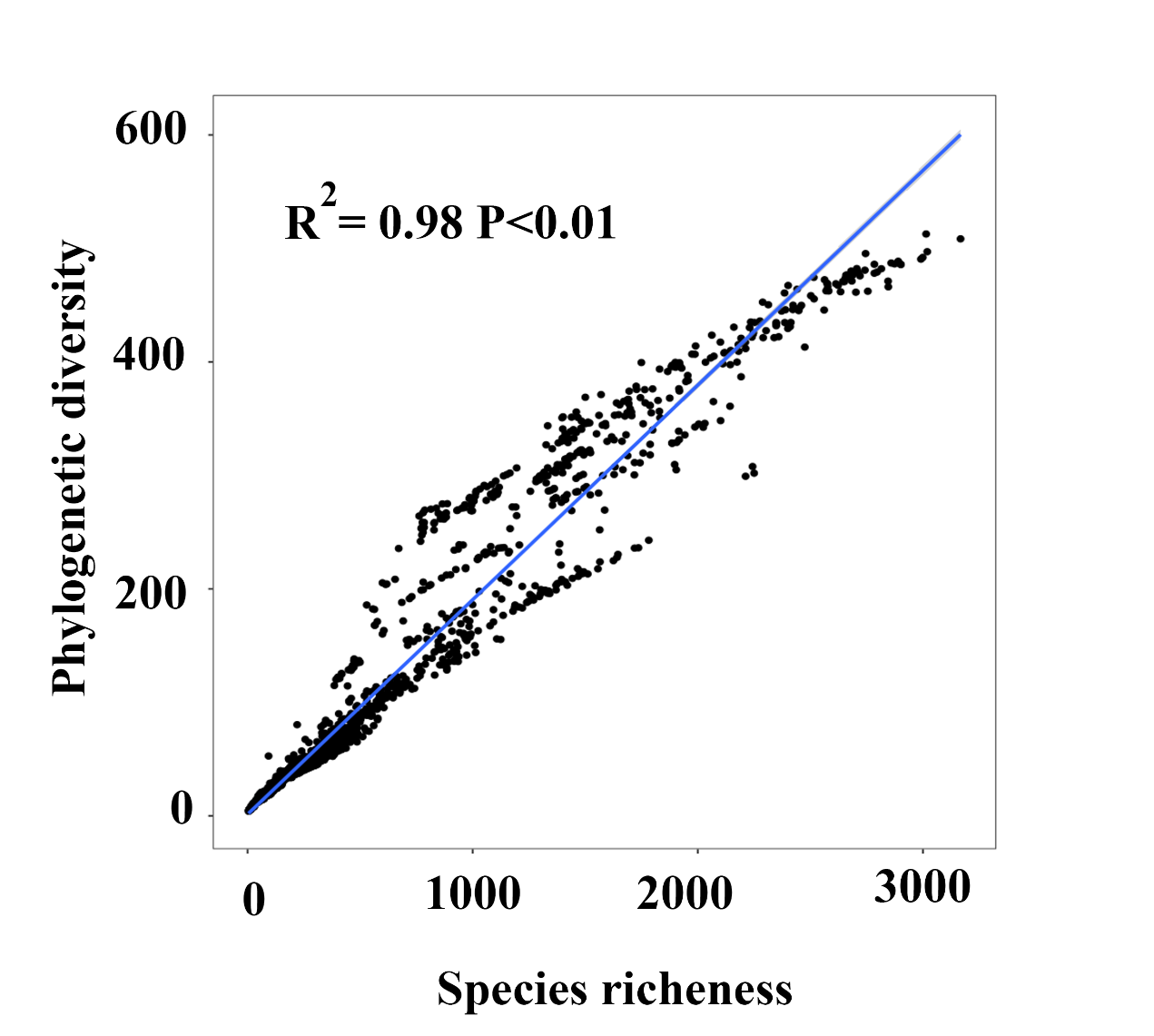


**Fig. S5 The spatial correlation tests between marine SR and PD.**


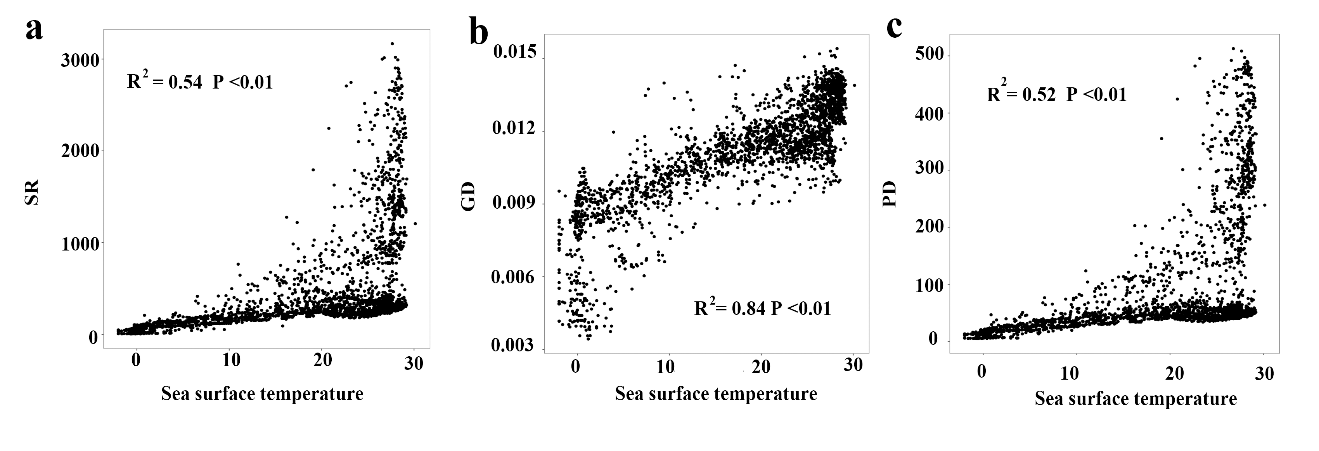


**Fig. S6 The spatial correlation tests between sea surface temperature and marine SR, GD and PD**. a) The spatial correlation tests between sea surface temperature and marine SR. b) The spatial correlation tests between sea surface temperature and marine GD. c) The spatial correlation tests between sea surface temperature and marine PD.

**
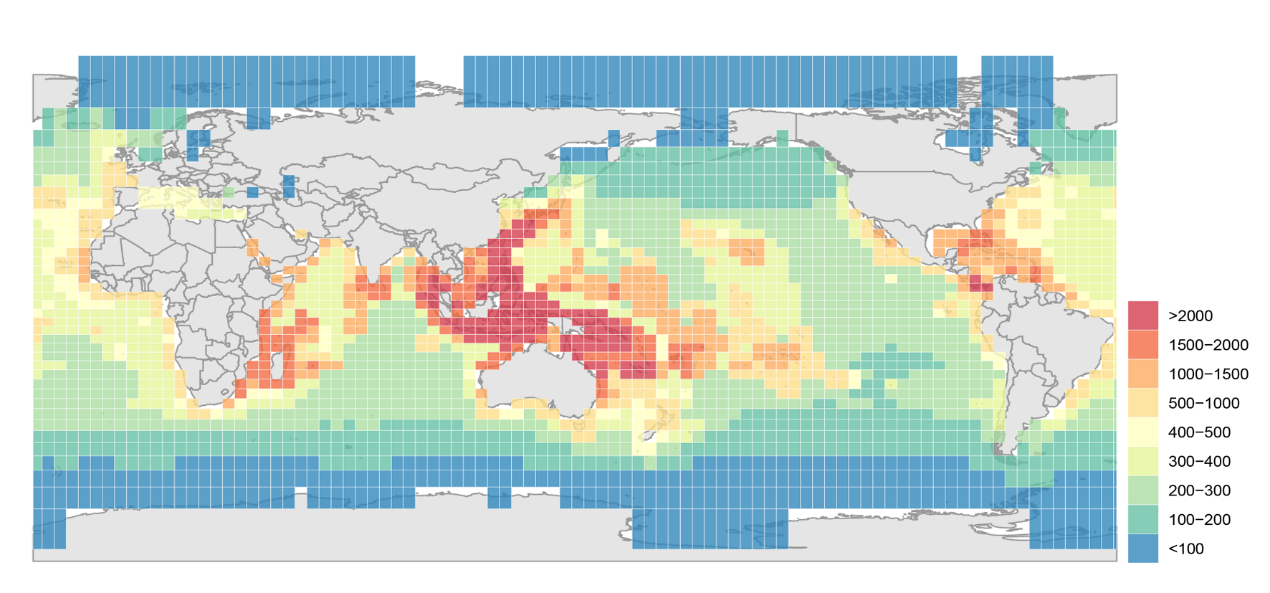
**

**Table S7. The global distribution pattern of marine species richness.**

**
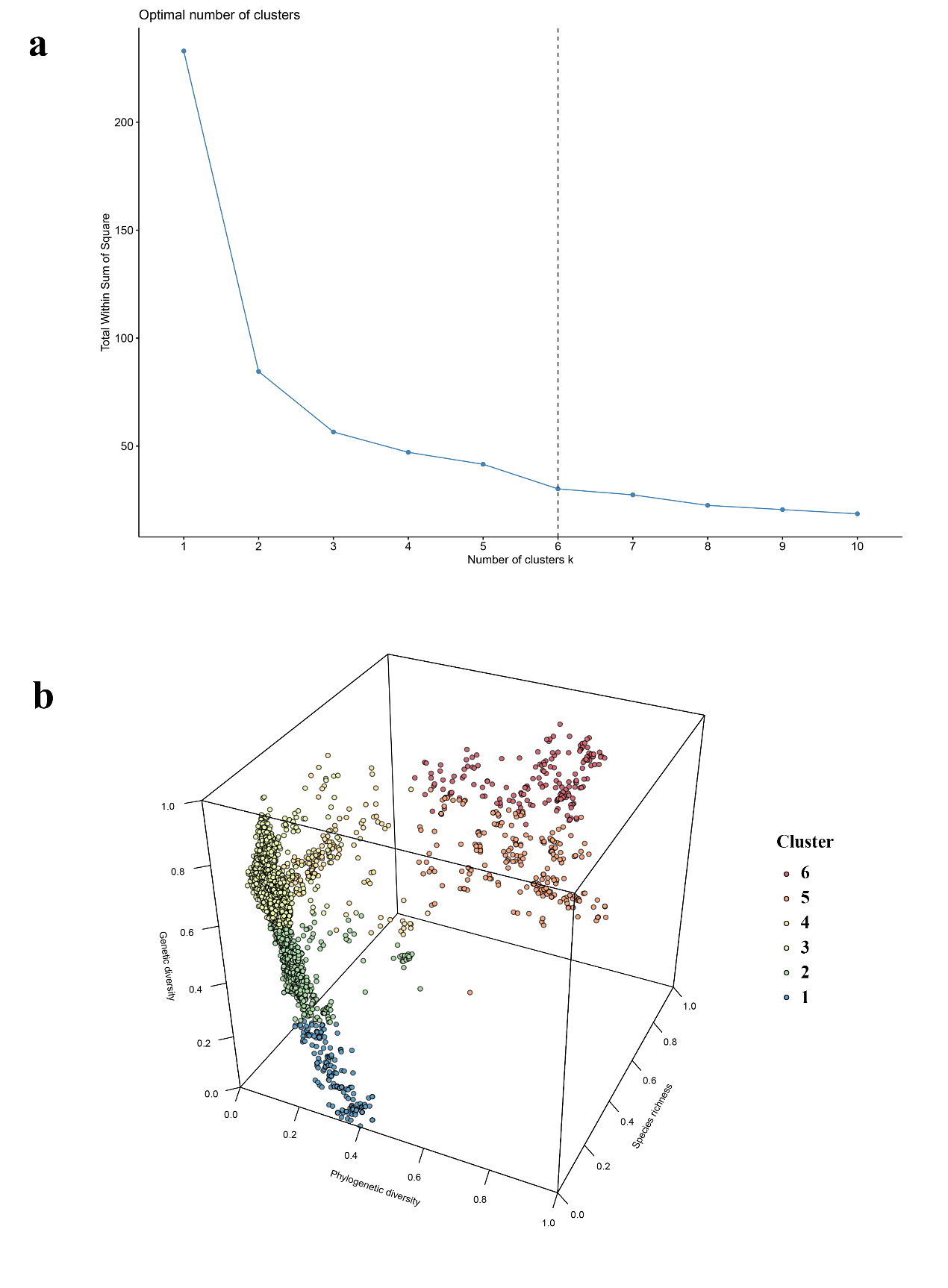
**

**Fig. S8. The number of probable clusters.** (a) The scree plots showing the optimal number of clusters. (b) The three-dimensions scatter plots of the grid cells based on normalized values of marine SR, GD and PD.

**
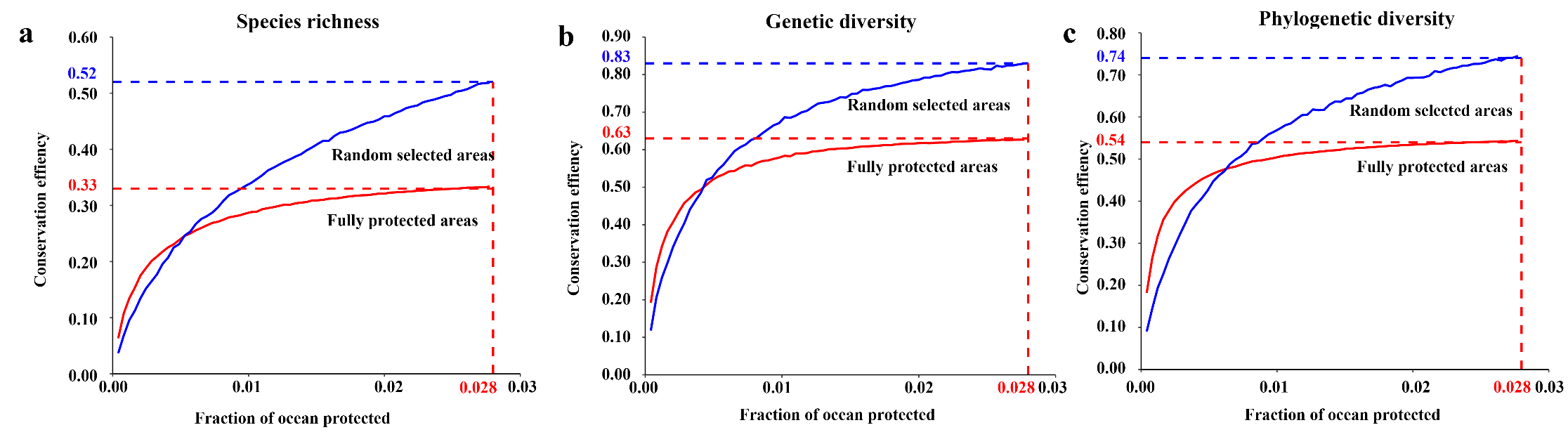
**

**Fig. S9.** The comparison of protection efficiency between fully protected areas and random selected areas for (a) SR (b) GD and (c) PD.

**
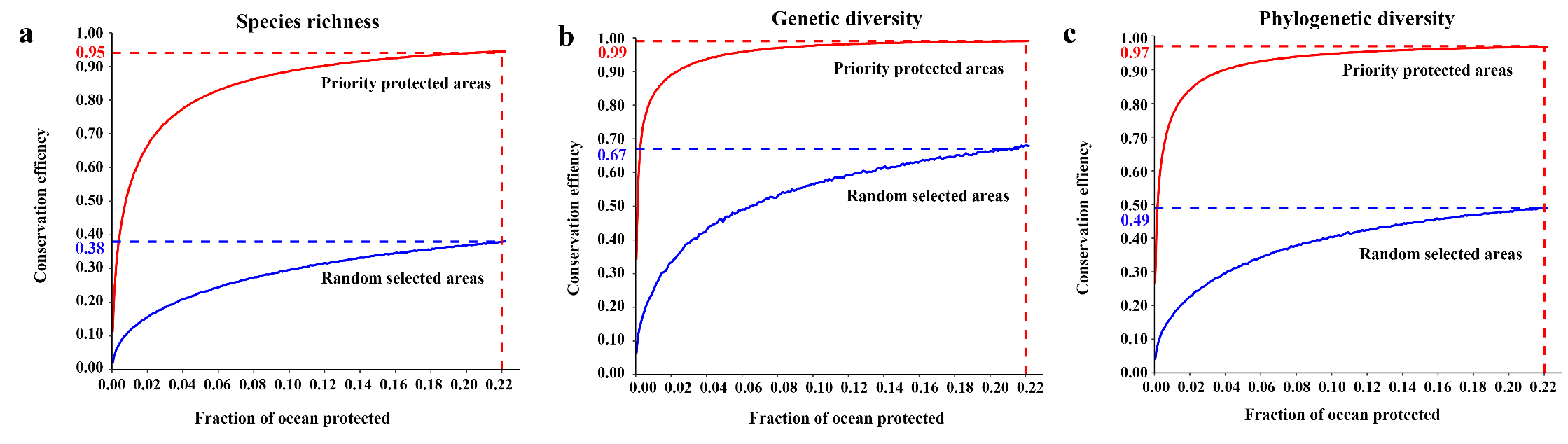
**

**Fig. S10.** The comparison of protection efficiency between priority protected areas and random selected areas for (a) SR (b) GD and (c) PD.

References for Supplementary Data

1. Miraldo A, Li S, Borregaard MK et al. An Anthropocene map of genetic diversity. *Science* 2016; 353(6307):1532-1535.
2. Dutilleul P, Clifford P, Richardson S, et al. Modifying the t test for assessing the correlation between two spatial processes. *Biometrics* 1993; 305-314.
3. Clifford P, Richardson S, Hemon D. Assessing the significance of the correlation between two spatial processes. *Biometrics* 1989;123-134.
4. Alò D, Lacy SN, Castillo A, et al. The macroecology of fish migration. Global Ecology and Biogeography 2020; 30: 99–116.
5. Faith DP. Conservation evaluation and phylogenetic diversity. *Biological Conservation* 1992; 61(1):1-10.
6. Procheş S, Wilson JR, Cowling RM. How much evolutionary history in a 10 x 10 m plot? Proceedings Biological sciences 2006;273(1590):1143-8.
7. Kembel SW, Cowan PD, Helmus MR, et al. Picante: R tools for integrating phylogenies and ecology. *Bioinformatics* 2010; 26(11):1463-1464.
8. Sbrocco EJ, Barber PH, MARSPEC: ocean climate layers for marine spatial ecology. *Ecology* 2013; 94(4), 979-979.
